# Qualities of an effective teacher: what do medical teachers think?

**DOI:** 10.1186/1472-6920-13-128

**Published:** 2013-09-17

**Authors:** Simerjit Singh, Dinker R Pai, Nirmal K Sinha, Avneet Kaur, Htoo Htoo Kyaw Soe, Ankur Barua

**Affiliations:** 1 Department of Orthopaedics, Melaka Manipal Medical College, Jalan Batu Hampar, Bukit Baru, Melaka, Malaysia; 2 Department of Surgery, Melaka Manipal Medical College, Jalan Batu Hampar, Bukit Baru, Melaka, Malaysia; 3 Department of Foundation in Science, Melaka Manipal Medical College, Jalan Batu Hampar, Bukit Baru, Melaka, Malaysia; 4 Department of Community Medicine, Melaka Manipal Medical College, Jalan Batu Hampar, Bukit Baru, Melaka, Malaysia; 5 Department of Community Medicine, International Medical University (IMU), No. 126, Jalan Jalil Perkasa 19, Bukit Jalil, 5700 Kuala Lumpur, Malaysia

**Keywords:** Medical teacher, Medical education, Effective teacher, Teacher perspectives

## Abstract

**Background:**

Effective teaching in medicine is essential to produce good quality doctors. A number of studies have attempted to identify the characteristics of an effective teacher. However, most of literature regarding an effective medical teacher includes student ratings or expert opinions. Furthermore, interdisciplinary studies for the same are even fewer. We did a cross-sectional study of the characteristics of effective teachers from their own perspective across medicine and dentistry disciplines.

**Methods:**

A questionnaire comprising of 24 statements relating to perceived qualities of effective teachers was prepared and used. The study population included the faculty of medicine and dentistry at the institution. Respondents were asked to mark their response to each statement based on a 5-point Likert scale ranging from strongly disagree to strongly agree. These statements were grouped these into four main subgroups, viz. Class room behaviour/instructional delivery, interaction with students, personal qualities and professional development, and analysed with respect to discipline, cultural background, gender and teaching experience using SPSS v 13.0. For bivariate analysis, t-test and one way ANOVA were used. Multiple linear regression for multivariate analysis was used to control confounding variables.

**Results:**

The top three desirable qualities of an effective teacher in our study were knowledge of subject, enthusiasm and communication skills. Faculty with longer teaching experienced ranked classroom behaviour/instructional delivery higher than their less experienced counterparts. There was no difference of perspectives based on cultural background, gender or discipline (medicine and dentistry).

**Conclusion:**

This study found that the faculty perspectives were similar, regardless of the discipline, gender and cultural background. Furthermore, on review of literature similar findings are seen in studies done in allied medical and non-medical fields. These findings support common teacher training programs for the teachers of all disciplines, rather than having separate training programs exclusively for medical teachers. Logistically, this would make it much easier to arrange such programs in universities or colleges with different faculties or disciplines.

## Background

Medical education is a constantly developing field. John T Biehn [1] stated that “the field of medical education is somewhat unusual in that the great majority of medical teachers have had no formal training as teachers. Most of us begin teaching careers with the naïve hope that we might impart something of value through having a combination of clinical experience and interest in the welfare of the student (or postgraduate trainee)”.

A number of studies have attempted to identify the characteristics of an effective teacher [2-4]. Sutkin et al. [5] listed five recurring features that the most effective teachers possess as “recognition of a relationship, emotional activation, generate responsibility, self-awareness and competence.” Non-cognitive qualities such as instilling self-confidence in students [6], encouraging creativity [7] along with fairness, empathy and humour [8] have been listed as important characteristics. For a long time the effectiveness of the teacher always has been measured on the basis of student outcomes or students’ perceptions [9-12]. Now the trend has changed to include the teacher’s self-evaluation or evaluation by peers in overall assessment of the medical teacher [13,14]. As teachers have a significant role in students learning, it is pertinent to identify the factors which make them effective [15].

Although many authors have published their personal opinions of characteristics of an effective teacher, fewer studies have used feedback from teachers themselves as compared to students’ ratings to define an effective teacher [13,16,17]. Sutkin et al. [5] in their literature review to identify specific characteristics of good medical teachers noted that these “characteristics were usually based on either the results of a survey of students/residents or the values or practical wisdom of the author(s).” Interdisciplinary studies of teacher effectiveness are further scarce [13]. Furthermore there is no agreement on evaluation of medical teacher effectiveness [18,19]. Most of the studies regarding teachers’ perceptions of effective teacher are either focussed on a specific subgroup of the medical profession like clinical skills lab [16] or surgical education [20], or have been done in non-medical or allied medical fields such as language [21], agriculture [22], business [23], nursing [24] and pharmacy [13]. Terese Stenfors-Hayes et al. [25] have said, “Most studies so far within this field have focused on teaching per se, whilst few focus on being a teacher”.

The aim of this study was to identify the relative importance of characteristics of effective teachers from medical teachers’ perspective. We have tried to make the study broad-based by including different medical disciplines (medicine and dentistry) and stratifying according to gender, cultural background and teaching experience.

## Methods

The study was conducted at Melaka Manipal Medical College (MMMC), Malaysia from March to June 2012. The participants comprised of faculty members of medicine undergraduate course (M.B.B.S.) and dental undergraduate course (B.D.S.). Approval from the college research and ethics committee was taken.

A cross-sectional study design was used. A questionnaire specifically prepared to meet the objective of the study was used. It was prepared by discussion among the authors after a review of the existing literature. Statements describing desired qualities of an effective teacher were formulated based on previous research [26-28]. The questionnaire was then refined and validated through a pilot study on a small subgroup (10) of faculty members and by obtaining inputs from medical education experts in the institution. Based on these inputs, the final questionnaire was then drafted and used in the study. Cronbach’s Alpha coefficient was calculated to measure the reliability of data form and was found to be 0.86.

All the faculty members of the institution (60) were approached to participate in the study on voluntary basis. Of these 57 responded. Written informed consent from the participants was taken. The questionnaire comprised of 24 statements relating to qualities of effective teachers. (Table 1) Each statement was discussed with the respondent to explain its meaning e.g. “teacher bias” addressed conscious/subconscious bias relating to gender, race, religion, ethnicity etc.; “Good communication skills” addressed diction, command over the language, method of delivery; “to be inspiring” described the quality of stimulating the student’s learning drive by generating interest in the subject. Similar clarification was made for all the statements. The statements were later clustered into four main subgroups according to their main thrust after discussion and consensus among authors, viz. classroom behaviour/instructional delivery, interaction with students, personal qualities and professional development parameters. The participants were asked to mark their response to each statement based on a 5-point Likert scale ranging from strongly disagree to strongly agree. In the same questionnaire data were also collected of their discipline, gender, academic designation, age, cultural background and teaching experience in years. Qualitative data were also collected in the form of one open ended question regarding any other quality of an effective teacher which they wanted to add. Anonymity was preserved during the data collection process.

**Table 1:** Distribution of respondents according to field, age, designation, gender, cultural background and teaching experience in years (n = 57)

**Variables**	**Frequency (%)**
**Discipline**	
Medicine	44 (77.2)
Dentistry	13 (22.8)
**Age(years)**	
29 – 39	21 (36.8)
40 – 49	16 (28.1)
≥ 50	20 (35.1)
Range = 29 - 73	
**Designation**	
Professor	17 (29.8)
Associate Professor	23 (40.4)
Assistant Professor	17 (29.8)
**Gender**	
Male	44 (77.2)
Female	13 (22.8)
**Cultural Background**	
Indian	33 (57.9)
Malaysian	10 (17.5)
Myanmar	9 (15.8)
Sri Lankan	5 (8.8)
**Teaching experience(years)**	
1 – 9	27 (47.4)
10 – 19	17 (29.8)
≥ 20	13 (22.8)
Range = 1 - 32	

The data collected were tabulated in Microsoft Excel 2010 and analysed using SPSS v 13.0. Mean and standard deviation were computed for quantitative variables while frequency and percentages were used to display qualitative variables. For bivariate analysis, t-test and one way ANOVA were used to find out association between dependent and independent variables. In this study, multiple linear regression was used for multivariate analysis to control confounding variables. Significant predictors and other variables which had a P-value <0.1 were entered into the multiple linear regression model. The Level of significance was set at 0.05.

## Results

Out of a total of 60 faculty members, 3 did not respond to the questionnaire and were excluded, giving a total of 57 (95%) faculty who participated in this study. Of these, 44 (77.2%) were from the field of Medicine and 13 (22.8%) were from the field of Dentistry. The age of the respondents ranged from 29 to 73 years (mean 45.5 ± 10.6 years). Designation wise, 40.4% were Associate Professor, 29.8% were Professor and the rest of 29.8% were Assistant Professor. More than two-third (77.2%) of the respondents were male. Based on cultural background, more than half (57.9%) were Indian, 17.5% were Malaysian, and 15.8% were Myanmar, while the remaining (8.8%) were Sri Lankan. Teaching experience ranged from 1 to 32 years, with a mean of 11.6 ± 7.6 years; nearly half of the respondents (47.4%) had experience of 1 – 9 years whereas 29.8% had 10 – 19 years and 22.8% had more than or equal to 20 years (Table 2).

**Table 2:** Mean score of Characteristics of Effective Medical Teachers in descending score order (n = 57)

**Categories**	**Variables**	**Mean score ± SD**
Classroom behaviour/ Instructional delivery	Should have good communication skills.	4.68 ± 0.54
	Should have good presentation skills.	4.46 ± 0.73
	Should use good sense of humor in teaching sessions.	3.91 ± 0.91
	Should be innovative in using technology in the classroom.	4.07 ± 0.90
	Should be well organized and possess excellent time management skills (good planner).	4.40 ± 0.82
	Should be inflexible regarding maintaining classroom discipline.	3.47 ± 1.25
Interaction with students/colleagues	Should be aware of students’ interests and needs.	4.12 ± 0.92
	Is easily approachable / affable.	4.23 ± 0.80
	Should not encourage student’s participation during theory lecture classes.	2.21 ± 1.38
	Should work well with colleagues and administrators.	4.26 ± 0.81
	Should be Inspiring & motivational to students.	4.51 ± 0.68
	Should be very generous in assessing the performance of the students during exams.	2.54 ± 1.38
	Should offer constructive criticism to the students.	4.31 ± 0.68
	Should trust & respect the students.	4.31 ± 0.80
	Should be caring and shows empathy towards students.	4.26 ± 0.69
Personal qualities	Should have leadership qualities.	2.05 ± 1.14
	Should be punctual.	3.47 ± 0.78
	Should be unbiased.	4.42 ± 0.98
	Should have sound knowledge of subject.	4.70 ± 0.53
	Should be enthusiastic and has passion to teach, enjoys teaching.	4.69 ± 0.54
	Should be honest, moral & ethical.	4.59 ± 0.73
Professional development	Should be up-to-date with the recent advancements in education technology	3.96 ± 1.08
	Should have publications and should be active in research.	3.29 ± 1.10
	Should be willing to learn & open to change (Flexible).	4.54 ± 0.59

In this study, characteristics pertaining to knowledge of subject was ranked highest (mean 4.70 ± 0.53), followed by enthusiasm/passion to teach (mean 4.69 ± 0.54) and communication skills (mean 4.68 ± 0.54) respectively. Mean scores of ‘Characteristics’ of effective medical teachers in are shown in Table 1.

Table 3 shows the comparison between the faculty’s perspective of an effective teacher in terms of each of the four sub-groups, (classroom behavior/instructional delivery, interaction with students/colleagues, personal qualities and personal development) and the general characteristics of the respondents. There were no significant differences in perspectives with regard to classroom behavior/instructional delivery (P-value = 0.426), interaction with students (P-value = 0.522), personal qualities (P-value = 0.687) and personal development (P-value = 0.059) between different disciplines (medical or dental) and gender. There were significant difference in perspectives of classroom behavior/instructional delivery between different cultural groups (P-value = 0.010), with Malaysians ranking it highest followed by Sri Lankans and Indians while Myanmar ranked it lowest. However, there were no significant differences in perspectives regarding interaction with students/colleagues (P-value = 0.060), personal qualities (P-value = 0.472) and personal development (P-value = 0.889) between teachers of different cultural backgrounds. There were significant differences of perspectives of classroom behavior/instructional delivery (P-value = 0.008) and interaction with students/colleagues (P-value = 0.027) between teachers with different years of experience, as the mean scores were higher with increase in years of experience. However, perspectives regarding personal qualities (P-value = 0.263) and personal development (P-value = 0.930) were not significantly different between teachers having different years of experience.

**Table 3:** Relationship between classroom behavior, interaction with students, personal qualities, personal development and general characteristics of the respondents (n = 57)

**Variables**	**N**	**Classroom Behavior**		**Interaction with students**		**Personal qualities**		**Personal development**	
		**Mean ± SD**	**P-value**	**Mean ± SD**	**P-value**	**Mean ± SD**	**P-value**	**Mean ± SD**	**P-value**
**Gender**^**ψ**^									
Male	44	31.89 ± 3.425	0.426	24.32 ± 3.226	0.522	30.57 ± 4.184	0.687	11.77 ± 2.291	0.059
Female	13	31.00 ± 3.764		23.69 ± 2.463		31.08 ± 3.121		12.62 ± 0.961	
Cultural background*									
Indian	33	31.30 ± 3.067	0.010	23.82 ± 3.036	0.060	30.24 ± 4.294	0.472	11.97 ± 0.395	0.889
Malaysian	10	34.00 ± 2.867		25.90 ± 2.846		31.90 ± 3.957		11.90 ± 0.690	
Myanmar	9	29.33 ± 3.873		22.67 ± 2.693		30.00 ± 3.240		12.33 ± 0.289	
Sri Lankan	5	33.80 ± 3.768		25.80 ± 2.775		32.40 ± 2.074		11.40 ± 1.166	
**Discipline**^**ψ**^									
Medicine	44	31.52 ±	0.525	24.11	0.782	30.50	0.522	11.84	0.414
Dentistry	13	32.23		24.38		31.31		12.38	
Experience in years*									
0 – 9	27	30.22 ± 3.434	0.008	23.07 ± 2.947	0.027	29.81 ± 4.658	0.263	12.04 ± 2.261	0.930
10 – 19	17	32.82 ± 3.321		24.88 ± 2.913		31.18 ± 3.358		12.00 ± 1.696	
≥ 20	13	33.23 ± 2.713		25.54 ± 2.847		31.85 ± 2.672		11.77 ± 2.315	

^Ψ^Independent sample T-test, *one way ANOVA.

Table 4 shows the linear regression analysis for classroom behavior/instructional delivery and interaction with students/colleagues. In this model, variables with P-value <0.1 were entered. Teaching experience in years and cultural background were the two variables thus considered for classroom behavior/instructional delivery and interaction with students/colleagues. After adjusting confounding variable, teaching experience in years was now found to be of only borderline significance with respect to classroom behaviour/instructional delivery (P-value 0.049), and there was no significant difference with respect to interaction with students. Cultural background was not significantly associated with classroom behavior/instructional delivery and interaction with students/colleagues.

**Table 4:** Multiple linear regression for classroom behavior/instructional delivery and interaction with students

**Variables**	**Classroom behavior/instructional delivery**			**Interaction with students**		
	**B**	**95% CI for B**	**P-value**	**B**	**95% CI for B**	**P-value**
Experience in years	1.303	0.008 – 2.597	0.049*	1.051	-0.102 – 2.205	0.073
Cultural background	0.412	-0.481 – 1.304	0.359	0.311	-0.485 – 1.106	0.437

In response to the open ended question, some other qualities of an effective teacher that were opined by few faculties were willingness to accept feedback, resource development, patience towards slow learners. Some suggested that teachers should not be over friendly with students and should be forgiving.

## Discussion

This study attempted to identify the relative importance of desired characteristics of an effective teacher according to the perspectives of medical teachers. In a teaching hospital, we usually serve the dual role of a clinician as well as a teacher. Most of us don’t have had any formal training as teachers. Nancy Hueppchen et al. [17] stated that most of the medical faculty learns to teach by observing their mentors or their teachers. This emphasizes the need to be aware of the qualities required to be an effective teacher to deliver the ever evolving medical curriculum.

In this study the ‘Knowledge of subject’ was rated the highest by the faculty irrespective of field, gender, teaching experience or cultural background. According to Adediwura and Tayo [29], it has been established that there is a high correlation between what teachers know and what they teach. Irby [30] identified six domains of knowledge essential for good medical teachers and stated “Excellence in clinical teaching requires clinical knowledge of medicine, of specific patients, and of context plus an educational knowledge of learners, general principles of teaching, and case-based teaching scripts.” Morrison et al. [31] in an online survey of residents and faculty using the Clinical Teaching Perception Inventory® (CTPI) included 28 descriptors of ideal clinical teachers and the top descriptors agreed between students and faculty were, “stimulating, encouraging, competent, communicates, and well-read.” Knowledge of subject is a cognitive quality which can be developed and has been found to be an important characteristic in various studies across non-medical disciplines also [32-35].

Enthusiasm was considered as the second most important parameter of an effective teacher in this study. This is similar to the findings of Yilmaz A [36], who noted that a teacher should be “Enthusiastic, excited about teaching, dynamic, and motivates students to learn”. Similarly in a study by Duvivier et al. [16] on perspectives of clinical skills lab teachers, enthusiasm emerged as the most important theme. In another study involving 214 nursing students, using professional competence, interpersonal relationship, personality characteristics and teaching ability as indicators, Tang, et al. [37] found that “personality related” features distinguished an effective teacher from an ineffective teacher and both effective and ineffective teachers were similar with respect to knowledge of subject. Two studies in the dentistry discipline found enthusiasm of the teacher as one of the important characteristic of an effective teacher [38,39].

The characteristic of communication skills was noted to be the third most desired trait for an effective teacher. In a study by Young and Shaw [10], effective communication skills emerged as one of the top seven qualities accounting for teaching effectiveness. Another study listed communications skills as a component of clinical teaching evaluation tool in the ambulatory setting [40]. Rider et al. [41] stated “Communication is a core clinical skill that can be taught and learned.” The fact that an effective teacher should have ‘leadership quality’ is well documented [3,42,43]. In contrast to this, in our study, the faculty perceived it to be least important. It is our belief that, a leader demands specific behaviour from his followers. This might be explained by the distinction between a “mentor”, which implies guidance and nurturing, and a leader, which implies an element of compulsion and disciplining. Hence the faculty might have felt that an effective teacher should be a guide more than a leader.

Based on factor subgroup analysis (Class room behaviour/Instructional delivery, Interaction with students/colleagues, Personal qualities and Professional development), faculty with longer teaching experience ranked ‘Class room behaviour/Instruction delivery’ higher than their less experienced counterparts. Experienced teachers believed that these traits are more important for achieving learning objectives. This is supported in a study conducted on music teachers in which the experienced teachers stressed on maintaining student behaviour/discipline more than their inexperienced counterparts [44]. However in the same study the experienced teachers ranked innovativeness lower than their less experienced counterparts. Even though we found that Malaysians ranked class room behaviour higher than their Myanmar counterparts, this can most likely be explained as Malaysian faculty in our institution were also the most senior, which became a confounding factor. Another interesting result was the response to the statement: “Should not encourage student’s participation during theory lecture classes”. One would have thought that everybody would strongly disagree here; instead the mean score for this parameter was a surprising 2.21 ± 1.38, i.e. they seem to be neutral. We feel that since this question was specific to ‘lecture’ as a teaching method, most faculties prefer not to be interrupted during the delivery of the lecture.

There are many limiting factors for this study. First, it is a single centre study hence the results cannot be generalized. Secondly, some of the subgroups were small in size. Thirdly, we use a new tool that needs further validation. Nevertheless, this study attempted to determine the effect of cultural background, gender, discipline and teaching experience on perspectives of effective teaching which to the best of our knowledge has been scarcely studied in medical field. Furthermore we noted similarity regarding the traits of effective teaching across medicine and various allied medical and non-medical disciplines. In future, we intend to establish concurrent validity of this tool by comparing with other validated tools such as the Cleveland Clinical Teaching Effectiveness Inventory and then use this tool for faculty evaluation and feedback so as the faculty can improve their teaching.

## Conclusion

Effective teaching in medicine is essential to produce good quality doctors. In the words of Ernest Leroy [45], “a poor surgeon hurts 1 person at a time but a poor teacher hurts 130”. Fewer studies have been done so far of medical teachers perspectives about effective teaching qualities compared to student ratings/surveys. In this study we examined if the perspectives of teachers in medical discipline regarding the qualities of an effective teacher are unique. However, we found that theses perceptions are similar, regardless of the discipline, cultural background and gender. This study findings support the justification for common teacher training programs for teachers of all discipline, rather than having separate training programs exclusively for medical teachers. Logistically, this would make it much easier to arrange such programs in universities or colleges with different faculties or disciplines.

## Competing interests

The authors declare that they have no competing interests.

## Authors’ contributions

SS and DP have contributed in conception and design of the study. NK, AK, HH and AB participated in acquisition of data, analysis and interpretation of data. SS, DP, AK and AB and drafted the manuscript. All authors contributed to revise it critically. All authors read and approved the final manuscript.

## Pre-publication history

The pre-publication history for this paper can be accessed here:

http://www.biomedcentral.com/1472-6920/13/128/prepub
